# Geostatistical COVID-19 infection risk maps for Portugal

**DOI:** 10.1186/s12942-020-00221-5

**Published:** 2020-07-06

**Authors:** Leonardo Azevedo, Maria João Pereira, Manuel C. Ribeiro, Amílcar Soares

**Affiliations:** CERENA, DECivil, Instituto Superior Técnico, Universidade de Lisboa, Av. Rovisco Pais, 1049-001 Lisbon, Portugal

**Keywords:** COVID-19, Disease mapping, Block direct sequential simulation, Infection risk maps

## Abstract

The rapid spread of the SARS-CoV-2 epidemic has simultaneous time and space dynamics. This behaviour results from a complex combination of factors, including social ones, which lead to significant differences in the evolution of the spatiotemporal pattern between and within countries. Usually, spatial smoothing techniques are used to map health outcomes, and rarely uncertainty of the spatial predictions are assessed. As an alternative, we propose to apply direct block sequential simulation to model the spatial distribution of the COVID-19 infection risk in mainland Portugal. Given the daily number of infection data provided by the Portuguese Directorate-General for Health, the daily updates of infection rates are calculated by municipality and used as experimental data in the geostatistical simulation. The model considers the uncertainty/error associated with the size of each municipality’s population. The calculation of daily updates of the infection risk maps results from the median model of one ensemble of 100 geostatistical realizations of daily updates of the infection risk. The ensemble of geostatistical realizations is also used to calculate the associated spatial uncertainty of the spatial prediction using the interquartile distance. The risk maps are updated daily and show the regions with greater risks of infection and the critical dynamics related to its development over time.

## Introduction

When the first case of human infection by the SARS-CoV-2 virus was reported in Wuhan city, Hubei Province, China, on 31 December 2019 [1], no one could predict its global effect. The contagion dynamics of the SARS-CoV-2 virus caught the scientific community and health systems by surprise, especially in respect of the rapid speed with which it spread. Lack of knowledge of SARS-CoV-2 and the disease it causes has resulted in the development of a range of strategies that seek to combat and mitigate COVID-19 while minimizing its economic impact [2–4]. The epidemic soon escalated into a global pandemic [5] that had serious implications in terms of fatalities, the stress on health systems and reduced economic activity, which is affecting everything while exacerbating existing economic and social inequalities [6–8].

During the early stages of the spread of a virus—when there are few infected people—environmental epidemiology using contagion risk models (e.g., SIR models [9]) is essential in informing and guiding public health officials and governments as they develop strategies designed to manage the crisis and avoid the breakdown of health systems [10, 11]. However, since contagion depends on individual and social behaviour, these models are difficult to calibrate at a relatively small-scale and their spatiotemporal predictions highly uncertain. Due to the exceptional propagation of COVID-19 contagion, governments adopted a range of strategies in an attempt to control contagion, ranging from mitigation to suppression. Whatever the strategy employed, the need to monitor and control the development of the disease remains in order to assess high-risk individuals, as the elderly, the effectiveness of the measures to prevent virus propagation during all stages of the pandemic, to manage the medical resources required to combat the disease and learn lessons ahead of any possible second and third outbreaks, while developing a vaccine. There several strategies to monitor and control the virus, such as monitoring deaths, syndromic surveillance or massification of tests in affected communities and at-risk populations to asses disease prevalence. In all cases, tools that help to understand spatial and temporal epidemic dynamics (e.g., https://covidmap.aledade.com/) are needed.

One of the traditional ways to map the risk of disease is through choropleth maps, which are usually available as rates (counts or proportions) of aggregated data by region or area (e.g., municipality boundaries). However, risks by area may change if the same data are aggregated using different spatial boundaries. One of the main contributions of geostatistics for the analysis of public health data is that it addresses the improvement of methods used to map the risk of disease. Geostatistical models have found ways to accommodate count data (or rates) attached to areal spatial supports that contribute to reducing the biased visual perception produced by choropleth maps and to facilitate the analysis of the relationships between risks measured over different spatial supports. The choropleth maps would have constant rates per municipalities and sharp discontinuities at the boundary of each municipality. On the other hand, geostatistical maps, as those proposed herein, do show spatial variability within each municipality and do not exhibit sharp discontinuities at the limits of each municipality as the spatial continuity pattern is imposed by the geostatistical method used.

In this context, an infection risk spatial model for COVID-19, which is based on a geostatistical framework, was proposed and implemented in mainland Portugal, considering the infection rate by municipality. The novelty of this approach is that the daily map is calculated from the number of confirmed positive tested cases reported by the Portuguese Directorate-General for Health (DGS, acronym in Portuguese) and made available publicly online.^1^ This study seeks to characterize the spatial dispersion of infection risk and attached uncertainty. It is a geostatistical simulation model that accounts for the infection risk uncertainty (i.e., error), derived by the population size of each municipality as reported by the Statistics Portugal [12]. The model outputs a set of possible scenarios from which we compute daily updates of local infection risk map and its uncertainty. At the same time, it facilitates monitoring and evaluating local infection risk dynamics. In addition, when decisions are made based on the risk map, such as the allocation of medical resources to prevent critical situations from arising, it is important to take into account the map’s uncertainty.

Next, we detail the geostatistical background related to the spatial modelling tool, direct block sequential simulation [13], used to map the COVID-19 infection risk in Portugal. We follow this with a brief description of the spatial continuity modelling and its importance within the simulation model. The last section summarizes the implementation of this case study.

## Methodology

The proposed model for mapping the COVID-19 infection risk in Portugal builds upon the Poisson model for rare diseases proposed by Waller and Gotway [14] and extended into a geostatistical framework by Goovaerts [15] and Oliveira et al. [16].

To predict the spatial distribution of the COVID-19 infection risk for a specific period and the associated uncertainty in mainland Portugal, we use a geostatistical model based on direct block sequential simulation (block-DSS; [13]), which accounts for the noise caused by spatial uncertain data as a function of population size and weights the estimation of the risk semivariogram accordingly.

Consider $c\left( {\varvec{u}_{\alpha } } \right)$, the number of infections notified (i.e., confirmed positive case tests) in each municipality $\alpha$ (with $\alpha = 1, \ldots ,N$ municipalities) since COVID-19 pandemic was declared up to a given day, referenced by its geometric centroid $\varvec{u}_{\alpha }$ with coordinates $\left( {x_{\alpha } ,y_{\alpha } } \right)$ and $n\left( {\varvec{u}_{\alpha } } \right)$, the size of the population at risk (i.e., resident population of a given municipality). The infection rate $z\left( {\varvec{u}_{\alpha } } \right)$ can be written as:

1 $$z\left( {\varvec{u}_{\alpha } } \right) = \frac{{c\left( {\varvec{u}_{\alpha } } \right)}}{{n\left( {\varvec{u}_{\alpha } } \right)}}.$$

One can assume that $z\left( {\varvec{u}_{\alpha } } \right)$, the infection rate estimated from confirmed test cases, is a realization of a random variable $Z\left( {\varvec{u}_{\alpha } } \right)$, the true infection rate. The expected value of $Z\left( {\varvec{u}_{\alpha } } \right)$, $E\left[ {Z\left( {\varvec{u}_{\alpha } } \right)} \right]$, provides therefore an estimate for the underlying risk of infection, $R\left( {\varvec{u}_{\alpha } } \right)$. However, Z is affected by the population size, such that the infection rate, for a given municipality with small population size (i.e., small infection rate denominator), will have high variance and consequently the confidence in the infection rate estimate is low. A solution to overcome this problem known as the small number problem [15] is achieved using a function (smoother), such is the case of the Poisson kriging method which stabilizes infection rates with high variance to provide a smoothed infection rate, and thus accessing the risk of infection $R$.

In the Poisson kriging model [15], the disease count $c\left( {\varvec{u}_{\alpha } } \right)$ at each location $\varvec{u}_{\alpha }$ is interpreted as a realization of a random variable $C\left( {\varvec{u}_{\alpha } } \right)$ that follows a Poisson distribution, with the parameter expected number of counts per unit of time. This parameter is the product of the population size, $n\left( {\varvec{u}_{\alpha } } \right)$, and the local risk, $R(\varvec{u}_{\alpha }$), with expected mean $m$. The expectation of risk at any location is equal to the expectation of the infection rate

2 $$E\left[ {Z\left( {\varvec{u}_{\alpha } } \right)} \right] = E\left[ {R\left( {\varvec{u}_{\alpha } } \right)} \right] = m$$

and the risk variance is equal to the infection rate variance minus a term related to the size of the population,

3 $$Var\left[ {Z\left( {\varvec{u}_{\alpha } } \right)} \right] = Var\left[ {R\left( {\varvec{u}_{\alpha } } \right)} \right] + E\left[ {R\left( {\varvec{u}_{\alpha } } \right)/n(\varvec{u}_{\alpha } )} \right] = \sigma_{R}^{2} + m/n\left( {\varvec{u}_{\alpha } } \right).$$

The purpose of this model for the COVID-19 risk map is to access the uncertainty of infection risk through a stochastic simulation methodology capable of integrating the different demographic size of each municipality and the uncertainty attached to the infection rate by population size.

### Block sequential simulation

The aim of this model is to generate high-resolution maps of infection risk based on recorded infection rates, and the associated spatial uncertainty. As the data are recorded in municipalities of different sizes and populations, they are interpreted as block support data. Block data refers to a non-point support data, which is generally referred to a volume but in this case of aggregated health data it refers to an area. In the application examples shown herein the spatial area refers to a municipality. The block sequential simulation algorithm gives the framework to deal with data with varying spatial support (i.e., varying size and shape of the municipality) and to make predictions with change of support. Thus, point and block support can be interpreted as two different support scales, in this case the scale related to the map cells can be referred as point support, because it denotes a small area when compared with the municipality areas. For sake of simplicity, we will refer to map cells as “point support” from this point forward.

In this sense, this means the resulting high-resolution risk maps are point support based. The stochastic simulation model is based on the work of Liu and Jounel [13] and Soares [17].

#### Estimation of local means of risk

At each step of the proposed stochastic simulation methodology [16], local means and variances of $Z\left( \varvec{x} \right)$ are assessed by block kriging: a kriging technique that accounts simultaneously for point and block data [13], where the block data, $B_{v} \left( {\varvec{u}_{\alpha } } \right)$, are defined as the spatial linear average of point values, $Z\left( {\varvec{u^{\prime}}} \right)$, within the block volume, which in this particular case simplifies to an area, $\varvec{v}$:

4 $$B_{v} \left( {{\mathbf{u}}_{\alpha } } \right) = \frac{1}{\left| v \right|}\mathop \smallint \limits_{v}^{{}} L_{\alpha } \left( {Z\left( {\varvec{u^{\prime}}} \right)} \right)d\varvec{u^{\prime}}\quad \quad \forall \alpha ,$$

where *L*_*α*_ is a known linear averaging function. The simple kriging estimator, $Z_{SK}^{*} \left( \varvec{u} \right)$, at any given location, $\varvec{u}_{\varvec{\alpha}}$, is conditioned to both point, $z\left( {\varvec{u}_{\alpha } } \right)$, and block data, $B_{v} \left( {\varvec{u}_{\beta } } \right)$:

5 $$z\left( \varvec{u} \right)^{*} - m = \mathop \sum \limits_{\alpha } \lambda_{\alpha } \left( {\varvec{u}_{\alpha } } \right) \cdot \left[ {z\left( {\varvec{u}_{\alpha } } \right) - m} \right] + \mathop \sum \limits_{\beta } \lambda_{\beta } \left( {\varvec{u}_{\beta } } \right) \cdot [B_{v} \left( {\varvec{u}_{\beta } } \right) - m ],$$

where $m_{0}$ is the stationary mean and the kriging weights $\lambda_{\alpha }$ and $\lambda_{\beta }$ are the solution of the linear kriging system:

6 $$\left[ {\begin{array}{*{20}c} {\lambda_{\alpha } } \\ {\lambda_{\beta } } \\ \end{array} } \right] = \left[ {\begin{array}{*{20}c} {{\mathbf{C}}_{PP'} {\bar{\mathbf{C}}}_{PB} } \\ {{\bar{\mathbf{C}}}_{PB}^{\text{t}} {\bar{\mathbf{C}}}_{{BB^{'} }} } \\ \end{array} } \right]^{ - 1} .\left[ {\begin{array}{*{20}c} {{\mathbf{C}}_{{PP_{0} }} } \\ {{\bar{\mathbf{C}}}_{{BP_{0} }} } \\ \end{array} } \right],$$

where ${\mathbf{C}}_{PP'}$, ${\bar{\mathbf{C}}}_{PB}$ and ${\bar{\mathbf{C}}}_{{BB^{'} }}$ are spatial covariances at point–point average point-block and average block–block [18] data supports, respectively. ${\mathbf{C}}_{{PP_{0} }}$ and ${\bar{\mathbf{C}}}_{{BP_{0} }}$ are spatial covariances between point support and average block support data and, the point support estimate in location, $\varvec{u}$, respectively. Please note that the kriging system assumes a second order stationary assumption, this means that the spatial covariances do not depend on data values but exclusively on the distance between their locations (i.e., the vector ${\mathbf{h}}$). Simplifying, the spatial covariance function can be derived as $C\left( {\mathbf{h}} \right) = C\left( 0 \right) - \gamma \left( {\mathbf{h}} \right)$, where $C\left( 0 \right)$ is the sill of the semivariogram model $\gamma \left( {\mathbf{h}} \right)$ fitted to a population-weighted semivariogram (see next section).

#### Integrating data uncertainty

As there is noise/uncertainty attached to infection rates, resulting from population size [15, 16], the population size can be used to quantify uncertainty through an ‘error variance’ term, $m^{*} /n\left( {{\mathbf{u}}_{{{\alpha }}} } \right)$, where $m^{*}$ is the population weighted mean, for zero distance covariances (Eq. 3), which can be introduced into the kriging system (Eq. 6), leading to what is called Poisson kriging [15].

Assuming block errors, say $r_{v}$, are homoscedastic and not cross-correlated, with zero mean and known variance, then the covariance between two errors located at $\varvec{u}_{\alpha } {\text{and }}\varvec{u}_{\beta }$ is:

7 $$C\left[ {r_{v} \left( {\varvec{u}_{\alpha } } \right),r_{v} \left( {\varvec{u}_{\beta } } \right)} \right] = \left\{ {\begin{array}{*{20}l} {\sigma_{R}^{2} \left( {\varvec{u}_{\alpha } } \right)} & {if} & {\varvec{u}_{\alpha } = \varvec{u}_{\beta } } \\ 0 & {if} & {\varvec{u}_{\alpha } \ne \varvec{u}_{\beta } } \\ \end{array} } \right..$$

Considering blocks $B$ and $B^{\prime}$, generically located at ${\mathbf{u}}_{\alpha }$ and ${\mathbf{u}}_{\beta }$. If errors are independent of the variable value, uncorrelated and with known variance, then:

8 $$C_{BB'} = \left\{ {\begin{array}{*{20}l} {C_{{BB^{\prime}}} \left( {\varvec{u}_{\alpha } ,\varvec{u}_{\beta } } \right) + \sigma_{R}^{2} \left( {\varvec{u}_{\alpha } } \right)} & {if} & {\varvec{u}_{\alpha } = \varvec{u}_{\beta } } \\ {C_{{BB^{\prime}}} \left( {\varvec{u}_{\alpha } ,\varvec{u}_{\beta } } \right) } & {if} & {\varvec{u}_{\alpha } \ne \varvec{u}_{\beta } } \\ \end{array} } \right..$$

#### Block sequential simulation

Block sequential simulation [13] is the extension of direct sequential simulation [17] by integrating different support data. The block sequential simulation algorithm workflow can be summarized as follows:

i) Define a random path that visits each node, $\varvec{u}$, of the simulation grid;
ii) For each node, $\varvec{u}$, search the conditioning data (closest original point data and previously simulated values and block data);
iii) Calculate the local covariance values: block-to-block, block-to-point, point-to-block and point-to-point; build and solve the block kriging system and obtain the local mean and variance kriging estimate at location $\varvec{u}$;
iv) Draw a value from the global probability distribution function centered at the local mean and bounded by the local variance obtained in (iii);
v) Add the simulated value to the data set and repeat steps (i) to (iv) until all grid nodes are simulated for one realization;
vi) Repeat steps (i) to (v) until a given pre-defined number of realizations are generated.

### Semivariogram of COVID-19 infection risk

As previously described, the block sequential simulation is based on a stationary spatial covariance model that reveals the main spatial continuity patterns of daily infection rates. These models are usually inferred from available experimental data: from the daily official infection rates.

As the experimental infection rates refer to different population sizes, these must be weighted differently when calculating the experimental variogram. In other words, municipalities with large populations should have greater weighting in the experimental variogram calculation. Here we applied an adaptation of the semivariogram proposed by [19, 20], which uses the weights, $w\left( \varvec{h} \right)$, to account for population size:

9 $$w\left( \varvec{h} \right) = \frac{{n\left( {\varvec{u}_{\alpha } } \right) n\left( {\varvec{u}_{\alpha } + h} \right)}}{{n\left( {\varvec{u}_{\alpha } } \right) + n\left( {\varvec{u}_{\alpha } + h} \right)}}.$$

The experimental semivariogram is calculated as follows [21]:

10 $$\gamma \left( \varvec{h} \right) = \frac{1}{{2\mathop \sum \nolimits_{\alpha = 1}^{{N\left( \varvec{h} \right)}} w\left( \varvec{h} \right)}}\mathop \sum \limits_{\alpha = 1}^{{N\left( \varvec{h} \right)}} \left\{ {w\left( \varvec{h} \right) \left[ {z\left( {\varvec{u}_{\alpha } } \right) - z\left( {\varvec{u}_{\alpha } + \varvec{h}} \right)} \right]^{2} } \right\},$$

where the vector, **h**, of each pair of municipality value was calculated with their centroids.

## Data: Daily updates of infection rates by municipality

The number of daily infections is provided for each municipality each day by the DGS. This number of confirmed tested COV 19 cases is then converted into infection rates, $z\left( {\varvec{u}_{\alpha } } \right)$, that consider the number of inhabitants per municipality (Eq. 1). In this model, the number of inhabitants refers to the estimates provided by the National Institute of Statistics in 2018 [12]. As for municipalities in which the number of confirmed infections is not made available, our model assumes all municipalities have at least two confirmed infections. This assumption is due to the fact that the DGS only publicly releases data of infection per municipality if this figure is above two, for the protection of individuals’ data. In this way, we are potentially overestimating the infection risk and producing slightly pessimistic scenarios regarding it in locations where we don’t have the “true” data value which coincides with locations where the risk is lower, but treating this municipalities has unknown data would even result in a larger overestimation. These data were also used to model the experimental variogram of the infection risk used in the direct block sequential simulation.

**Fig. 1:**
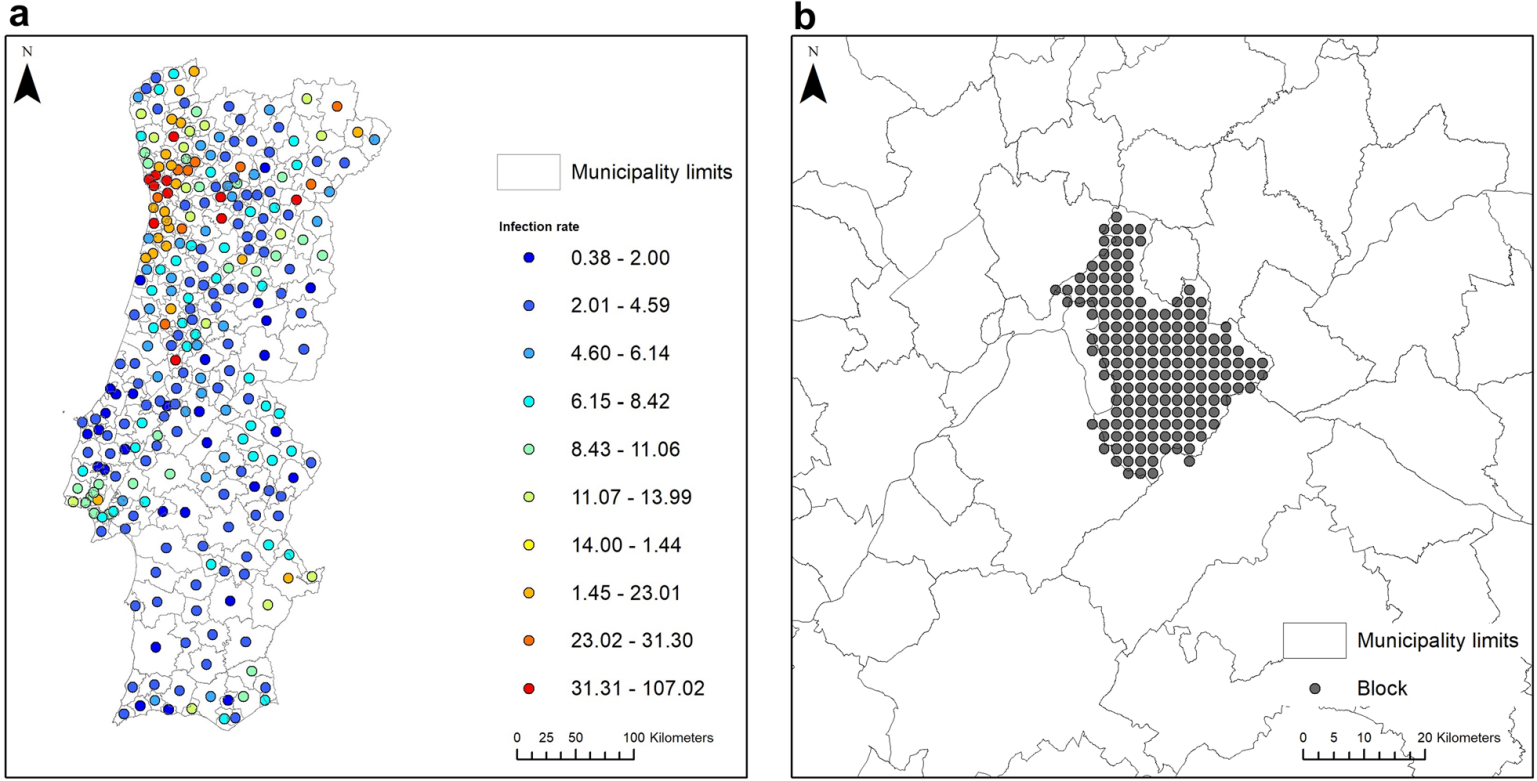
**a** Location of the centroid of each municipality in mainland Portugal and the infection rate by 10,000 inhabitants on 12 April 2020; **b** example of the regular discretization of a given municipality represented by the grey circles

Infection rates are assigned as point experimental data to the centroid of the municipality area (Fig. 1a). Each municipality area is discretized by a regular grid of points to define the block/areal data (Fig. 1b).

## Results

### Spatial continuity patterns analysis of COVID-19 infection rates

**Fig. 2:**
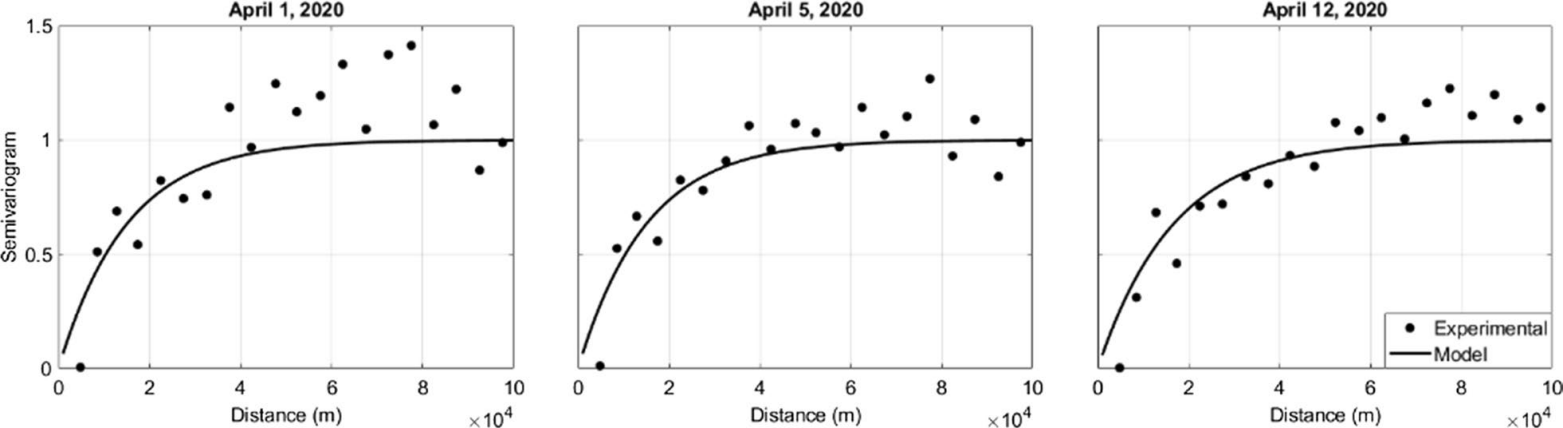
Experimental and variogram models of COVID-19 infection rate on three different days

**Fig. 3:**
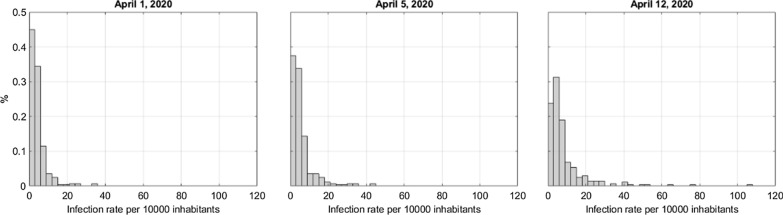
Histogram of confirmed COVID-19 infection on three different days, showing the increase in the number of infections over time

The experimental infection rate variograms, weighted for each population, were calculated according to Eqs. 9 and 10. Figure 2 shows the omnidirectional variograms for three different days, with the respective fitted model: a spherical model with a range of 60 km. The three variograms are standardized by the variance of the corresponding day. Figure 3 shows the infection rate histograms for the complete set of municipalities.

### COVID-19 infection risk maps

**Fig. 4:**
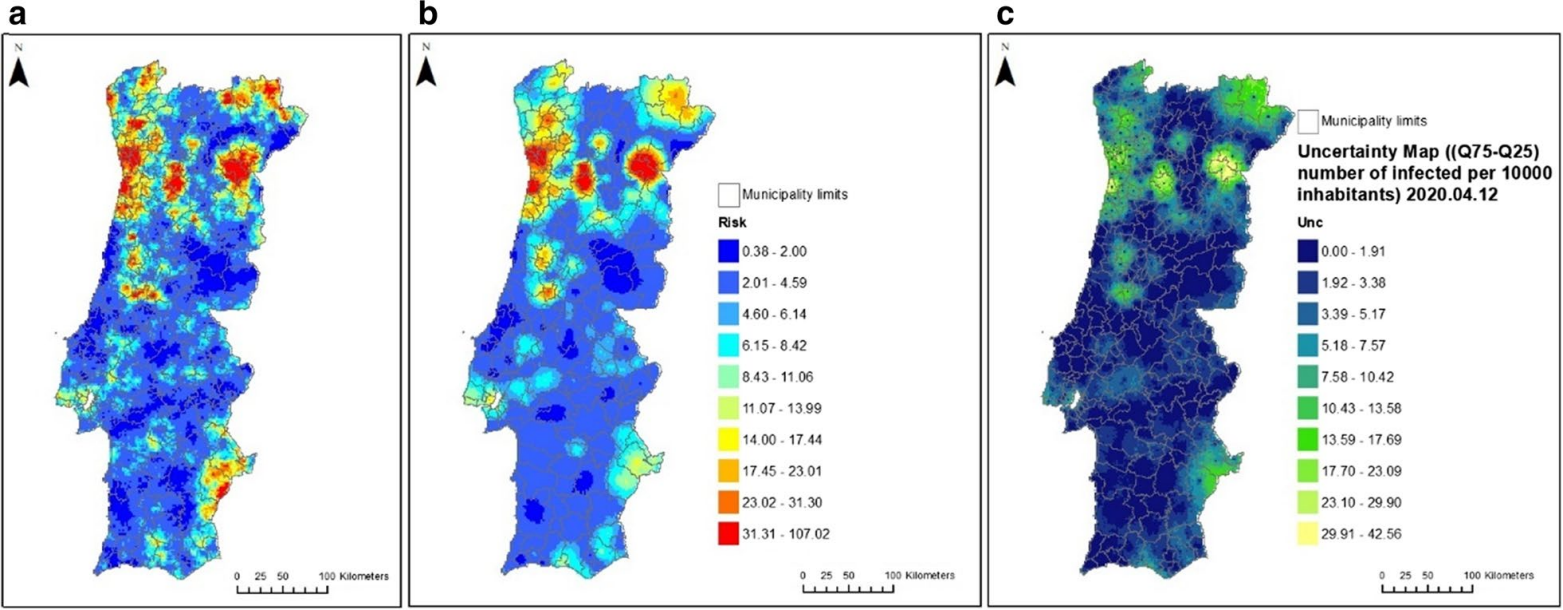
**a** Realization of infection risk; **b** median model of the infection risk by COVID-19 in mainland Portugal on April 12, 2020; and **c** the interquartile distance (Q3 - Q1) computed from a set of 100 realizations using the proposed method

The main output of the model presented here is a set of realizations of the spatial dispersion of COVID-19 infection risk in a regular grid of points (2 × 2 km) covering the whole country. Each realization (one example is shown in Fig. 4a) reproduces the experimental infection rate data assigned to the municipality’s geometric centre, reproducing the main spatial patterns as revealed by the spatial covariances and the main rate data statistics (histograms).

The ensemble of realizations can lead to two main high-resolution output maps. The median map of COVID-19 infection risk and the ensemble of realizations (Fig. 4b). The uncertainty attached to COVID-19 infection risk can be revealed by the variance or interquartile maps (Fig. 4c). This local uncertainty is related to the size of the municipality’s population. In this application example we used a set of one hundred high-resolution stochastic simulations.

The set of median models and uncertainty COVID-19 infection risk maps for a seven-day period are available in an interactive web-based application (http://cerena.ist.utl.pt/news/daily-infection-risk-maps-covid-19-portugal).

### Temporal trend of COVID-19 infection risk

**Fig. 5:**
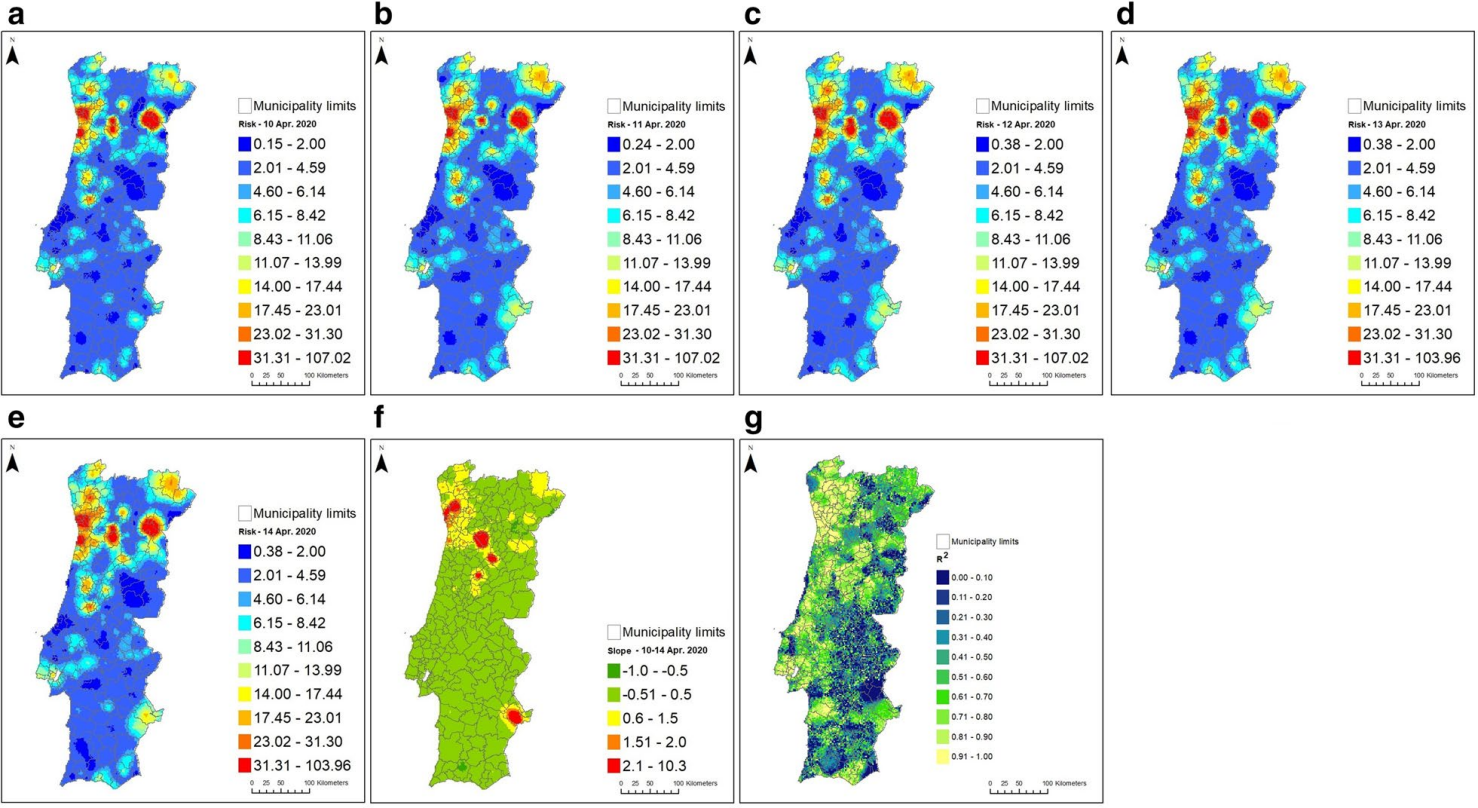
Median models of infection risk for: **a** April 10, 2020; **b** April 11, 2020; **c** April 12, 2020; **d** April 13, 2020; **e** April 14, 2020; **f** slope of linear regression of the median risk maps for five consecutive days; **g**$R^{2}$ map of the linear regression

One of the most important characteristics of the model presented here is its ability to provide an analysis of the infection spatial dynamic over time. This allows the proposed model to be part of a COVID-19 local infection evolution management tool. Several statistics on the local dynamics spatial infection patterns can be obtained: for example, the resulting ensemble of risk maps covering a period of several days or weeks may reveal a trend in the prevalence of infection over time. Assuming the linear behaviour of infection risk over a short period (for example, the median risk models obtained for five consecutive days in Fig. 5a–e), the linear regression slope is used as an indicator of the direction and intensity of the risk trend over time (Fig. 5f). The slope value in the legend indicates the increase and stability of the reduced level of risk on each day: slope = 1 means an increase of one infected/10,000 inhabitants per day; slope ≈ 0 shows no significant changes; slope = - 1 means a reduction of one infected/10,000 inhabitants. The uncertainty in the regression slope is shown in Fig. 5g by mapping the $R^{2}$ of the linear regression.

## Final remarks

The model proposed here is based on the stationarity assumptions of infection rate statistics: the global mean and variogram, and the reported infection rate stationarity for each municipality. When a given infection rate is reported for a municipality, a spatial stationarity of that rate is assumed for the entire municipality without taking population dispersion into account. Cluster infection data (occurring, for example, in retirement communities) can eventually skew the municipality’s infection rate, particularly when the municipality has a small population.

One of the most innovative components introduced by this model is its ability to be updated in the short-term. Thus, risk maps are produced using information provided daily by the DGS. Eventual sampling errors of the infected people in daily reports will be reflected in the maps.

## Conclusions

The proposed model for characterizing the COVID-19 infection risk, based on stochastic simulations, produced consistent results for risk and associated uncertainty. The daily update of risk and uncertainty maps enable a regional analysis of the dynamics of the phenomenon over time through, for example, the use of simple statistics, such as the slope of a short-term linear regression or a more comprehensive methodology such as functional data analysis [22]. The spatial uncertainty assessment of the model is an important feature of the algorithm, since it provides additional information about the local estimates to the decision process. For instance, the local probabilities of risk exceedance of a certain threshold can be computed from the simulated scenarios. However, the most significant impact of this model is its adoption as an instrument for managing the COVID-19 infection phenomenon, to be used together with other models and other relevant information, allowing health authorities to determine greater or lesser containment strategies and the establishment of lockdowns targeting specific areas where the infection risk is higher and increasing for time.

## Data Availability

Data and material are available upon request. Results of the method are available online: https://cerena.pt/news/daily-infection-risk-maps-covid-19-portugal.

## Footnotes

1 https://cerena.pt/news/daily-infection-risk-maps-covid-19-portugal.
